# Representing vaccine misinformation using ontologies

**DOI:** 10.1186/s13326-018-0190-0

**Published:** 2018-08-31

**Authors:** Muhammad Amith, Cui Tao

**Affiliations:** School of Biomedical Informatics, The University of Texas Health Science Center, 7000 Fannin Street, Suite 600, Houston, TX USA

**Keywords:** Vaccine, Misinformation, Ontology, Natural language processing, Semantic web, Semantic similarity, Microattribution

## Abstract

**Background:**

In this paper, we discuss the design and development of a formal ontology to describe misinformation about vaccines. Vaccine misinformation is one of the drivers leading to vaccine hesitancy in patients. While there are various levels of vaccine hesitancy to combat and specific interventions to address those levels, it is important to have tools that help researchers understand this problem. With an ontology, not only can we collect and analyze varied misunderstandings about vaccines, but we can also develop tools that can provide informatics solutions.

**Results:**

We developed the Vaccine Misinformation Ontology (VAXMO) that extends the Misinformation Ontology and links to the nanopublication Resource Description Framework (RDF) model for false assertions of vaccines. Preliminary assessment using semiotic evaluation metrics indicated adequate quality for our ontology. We outlined and demonstrated proposed uses of the ontology to detect and understand anti-vaccine information.

**Conclusion:**

We surmised that VAXMO and its proposed use cases can support tools and technology that can pave the way for vaccine misinformation detection and analysis. Using an ontology, we can formally structure knowledge for machines and software to better understand the vaccine misinformation domain.

## Background

Since their introduction, vaccines have been an important breakthrough that has led to the near-eradication of many infectious diseases. Some of these diseases include polio, typhoid, and smallpox - all which are now uncommon. But in the modern era, certain sectors of society have embraced a post-modernist approach that endorses “that science and ’experts’ are open to questioning... put[ting] greater emphasis on intuition and social relationships and tends to distrust the scientific method as the best paths to healing our ills” [1]. This, compounded with various other factors including misinformation about vaccines, has presented a problem in vaccine uptake into the population. The effects of this are troublesome, considering in one poll 20% of those surveyed believed that there is a link between autism and vaccine [2], in a Gallup poll, 58% are either unsure or actually believe that vaccines cause autism [3], and 11% presume that vaccines are not necessary and 25% presume that autism is a side-effect of vaccines in another survey of parents [4].

Vaccine skepticism dates back as far as the 19th century, when the United Kingdom introduced the Vaccination Act of 1853 requiring compulsory inoculation of children. Backlash to the law emerged with the formation of the Anti-Compulsory Vaccination League and ensuing publications to advocate anti-vaccination beliefs and ideas [5, 6]. In the 20th century, the retracted study by Andrew Wakefield that claimed a link between vaccine and autism had an unfortunate impact on vaccine discourse and the decline of MMR vaccine rates in certain regions of the world [7, 8]. Even to this day, Andrew Wakefield is still propagating the same discredited vaccine claims, and also has directed a documentary called “Vaxxed:From Cover-Up to Catastrophe” that received a special screening at the Cannes Film Festival [9]. Other figures, like U.S. President Donald Trump [10], Robert Kennedy, Jr of the Kennedy family [11], Dr. Robert Sears [12], Alex Jones [13], Bill Maher [14], Jenny McCarthy [15, 16], etc., have continued to express distorted claims about vaccines.

In the information age, the unregulated nature of the Web has provided free discourse and information sharing to anyone with a computer and Internet access. To some researchers, the Web is a “Pandora’s Box” that has both benefits and costs [17, 18], particularly its impact on health-seeking knowledge. In a Pew Research poll from 2013 [19], a majority of those surveyed (73%) sought health-related information with a third of those (35%) diagnosing themselves as opposed to seeing a doctor. In the same study, of the individuals who sought vaccine information (17%), 70% made a decision about vaccination based on the information they found. This may be troubling, as previous studies have highlighted that anti-vaccination websites appear highly ranked in search engine hits [17, 20]. Additionally, social media platforms have a significant impact on vaccination attitudes [17, 21–24]. Overall, the proliferation of vaccine misinformation is accessible to anyone with a mobile device and limited time to perform extensive research.

There are previous studies that have looked at the content of vaccine misinformation and motivation, but none that have investigated informatics tools that can assist and automate the analysis of vaccine misinformation to understand the drivers behind these false notions. The theoretical benefit of such tools can help process massive amount of content (i.e. social media posts), and also discover new knowledge that may not be apparent through manual human analysis. Numerous previous studies can help inform the development of tools and technology to accomplish this objective.

We aimed to use semantic web and ontological technology to represent the domain scope of vaccine misinformation. Also, with ontological representation, we intended to use this artifact to store various misconceptions about vaccines. This would eventually assist in a catalogue misinformation that can be queried and analyzed for future research. While some vaccines are associated with specific misinformation, we focused in this study on the general domain. The Vaccine Misinformation Ontology (VAXMO) is composed of existing ontologies - Misinformation Ontology and nanopublications - and is extended with features pertinent to the anti-vaccine domain. Lastly, we introduced possible use cases that will involve the vaccine misinformation ontology to identify misinformation for text-mining tasks and other applications.

## Semantic web and ontologies

The word ontology has its roots in metaphysical philosophy, extending back to Aristotle’s Categories, as a “nature of being”. In the early 90s, the definition of ontology was applied in the computer science field as a “specification of a conceptualization.” [25]. At the turn of the century, Sir Tim Berners-Lee described his vision for the next generation web called the “semantic web” in Scientific America, where ontologies would be the foundation for this vision [26]. Simply, an ontology is a machine-readable artifact that encodes a logical representation of a domain space using vocabularies, and their semantic meanings. It is the output of a knowledge engineering process where tools and methods are used to build the ontology [27]. Overall, ontologies are used for representing information and knowledge [28–30].

In general, knowledge in an ontology is represented as triple which is information presented in *subject*>*predicate*>*object*. Essentially, the *subject*>*predicate*>*object* are concepts that are “smallest, unambiguous unit of thought... [that are] uniquely identifiable” [31]. Each triple can seamlessly link to another triple to form an ontological knowledge-base. For this knowledge to be readable by a machine, we use a computer-based syntax to encode this knowledge. Once encoded, this artifact can be shared and distributed for various purposes. Moreover, using Web Ontology Language (OWL) or Resource Description Framework (RDF), a specific type of web ontology language syntax for ontologies, we can define more complex axioms and assertions to fully describe concepts which provide machine reasoning capabilities.

### Nanopublication primer

Semantic web technologies, specifically ontologies, have had continued impact on research and knowledge sharing, and standardization in the biomedical domain. Some of what has been described were the benefits of formalizing information, information integration, information reuse, and querying and search, etc. We introduce the use of nanopublication, which is an ontology-based micro-publishing format for encoding and distributing singular units of assertions. Nanopublications have been used primarily in the life sciences, pharma sciences, as well as genomics and proteomic research data [32]. The benefit of nanopublications include [32]:

- Improve finding of scientific information
- Connect scientific information from multiple sources
- Organize provenance information of the research finding
- Verifiable
- Small

The model or structure of a nanopublication involves a scientific assertion, provenance of the assertion, and provenance information of the nanopublication itself [33]. The scientific assertion component is the singular atomic finding that is represented as *subject*>*predicate*>*object*. An example would be “trastuzumab [subject] is indicated for (treats)[predicate] breast cancer[object]”. The other component is the provenance of the assertion, or “the origin or source of something” [34], which will express metadata information, like DOI, authors, research institution, time and date, experimental method, etc. The third part is the provenance information about the nanopublication, which generally indicates who created the nanopublication and when it was created (analogous to citation metadata).

Provided (Listing 1) is a basic example of a nanopublication encoding for the research assertion, “trastuzumab is indicated for (treats) breast cancer.” Specific discussion of the encoding is outside the scope of this proposal, and many references exist to provide further information [33, 35]. But briefly, the research assertion is coded in lines 14-16. Lines 18-22 provides provenance of the assertion - the time it was generated, the experiment it was derived from, and who conducted the experiment. Lines 24-27 provide information on the author of the nanopublication and when it was generated. Like all ontology-related artifacts, a unique identifier is associated with the nanopublication in lines 1-2.


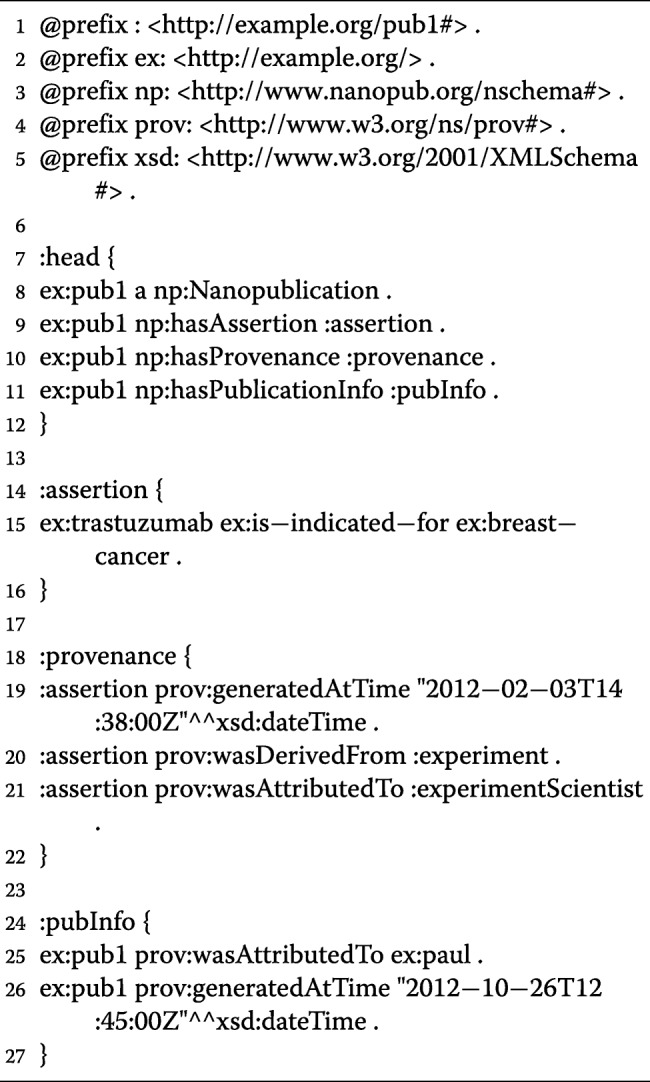


Like any ontological representation, many nanopublications that convey the same information can be aggregated and collated to form a singular machine-encoded statement called “S-Evidence” [31]. From a research point of view, the aggregation of similar research findings from different sources and authors can strengthen the trustworthiness of the finding. At the same time, each nanopublication with its own unique identifier can still be queried, or be utilized for any machine reasoning purposes [31].

## Methods

### VAXMO: Vaccine Misinformation Ontology

We designed and developed the Vaccine Misinformation Ontology (VAXMO) that models concepts pertaining to vaccine misinformation, and a schema that permits archiving of vaccine myths and misinformation. The foundation of VAXMO is built upon the work of Zhou and Zhang, who developed an ontology for general misinformation [36, 37]. The goal of their work was to “provide guidance to researchers on misinformation understanding, identification, and detection”, and it also considers the Information Theory model to derive concepts, and existing literature of misinformation. In addition to Zhou and Zhang’s Misinformation Ontology (MO), we also harnessed the use of the nanopublication format to store vaccine “theories” and their origin information. In the subsequent sections, we will summarize the main concepts for VAXMO model.

**Fig. 1:**
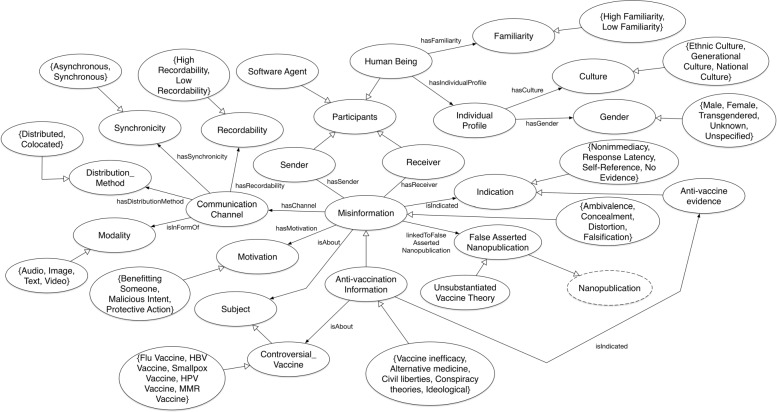
Class diagram of Vaccine Misinformation Ontology (VAXMO)

Figure 1 illustrates the class level description of the VAXMO ontology with extensions for anti-vaccination concepts. As noted earlier, the foundational concepts of the model are derived from Misinformation Ontology. At the time of this research, the OWL-based ontology of MO is not available on the web, so based on their early publications, we reconstructed the ontology in OWL2 with Protégé [38], and incorporated modifications to elaborate on the model. Zhou and Zhang [36, 37] provides theoretical detail on the misinformation concepts.

The central concept for VAXMO is *Anti-vaccination Information* which is a subclass of the *Misinformation* concept from MO. In addition to the subclasses for Misinformation (*Ambivalence*, *Concealment*, *Distortion*, and *Falsification*), *Anti-vaccination Information* concept introduces subclasses of itself - *Vaccine inefficacy*, *Alternative medicine*, *Civil liberties*, *Conspiracy theories*, *Falsehoods*, and *Ideological*. These subclasses for *Anti-vaccination Information* are based on classification of misinformation and myths from [17]. For the time being, some of the subclasses have not been extensively defined and may be equivalent or subcategories of the four subclasses for the *Misinformation* concept. While *Falsehood* may be the same as *Falsification*, but *Alternative medicine* might be equivalent to *Distortion* or *Conspiracy theories* to *Concealment*.

From Information Theory, the transmission of information encapsulates a sender and receiver. We represented the concept *Participants*, which permits defining a number of participants who are part of the misinformation process, and is a parent class of *Sender* and *Receiver* class. The *Anti-vaccination Information* inherits relationships to a *Sender* and *Receiver* from the original *Misinformation* concept. *Software Agent* and *Human Being* are subclasses of the *Participant*. *Human Being* is defined with an *Individual Profile* concept class that describes demographic information (*Culture* and *Gender* concept). *Human Being* has definitions that describes how familiar via the *Familiarity* class that the human participant(s) is with the misinformation.

Additionally, VAXMO associates *Anti-vaccination Information* concept with the *Communication Channel*. The *Communication Channel* represents how, when, and where misinformation is transmitted. This is depicted by concepts like *Availability*, *Synchronicity*, *Distribution Method*, and *Modality* classes - classes originating from MO. Also, *Anti-vaccination Information* has a property associated with *Controversial Vaccine* (a subclass of *Subject*) that defines what the *Anti-vaccination Information* class is referring to. In this specific domain, *Anti-vaccination Information* is about the vaccine topic (*Controversial Vaccine* concept). The *Controversial Vaccine* concept is further broken into subclasses pertaining to specific type of vaccines (e.g. *HPV Vaccine*, *MMR Vaccine*, etc.).

Both *Motivation* and *Evidence* are concepts described in VAXMO and are properties associated with *Anti-vaccination Information*. *Motivation* concerns the reason for transmitting misinformation (*Benefiting Someone*, *Malicious Intent*, *Protective Action*). *Evidence* is a class for conceptualizing supporting information.

**Fig. 2:**
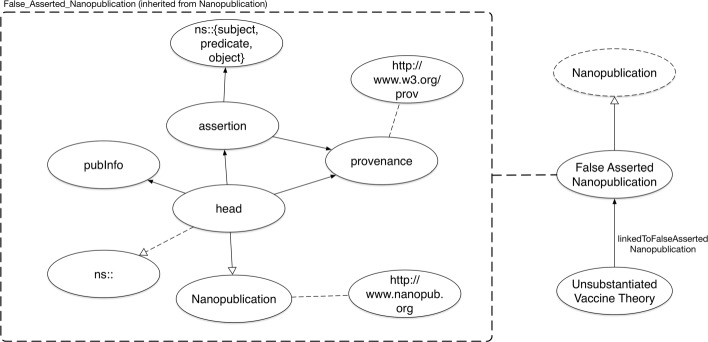
Excerpt of VAXMO’s nanopublication usage

For the purpose of collecting vaccine misinformation in the form of triples (e.g. *vaccines*>*causes*>*seizures*), we look to the nanopublication format. In order to model these triples belonging to a single concept, we extended it using the nanopublication graph model which was originally designed to encode scientific assertions in the form of triples. *False Asserted Nanopublication* class serves as a listing denoting exactly what the misinformation content is. We subclassed *Unsubstantiated Vaccine Theory* from *False Asserted Nanopublication* which is a subclass of nanopublication to inherit its graph model to represent the claims about vaccines. We view these claims as singular decomposed statements in the form of *subject*>*predicate*>*object*. Shown in Fig. 2, the nanopublication instance is associated with *Unsubstantiated Vaccine Theory*. This provides VAXMO with a means of cataloging samples of vaccine misinformation.

Lastly, to model cues associated with anti-vaccination misinformation, VAXMO modeled a relationship between *Anti-vaccination Information* with class *Anti-Vaccination Evidence* (*Indication*) that represents evidence associated with vaccine misinformation.

## Results

### Preliminary evaluation metrics

The Vaccine Misinformation Ontology (VAXMO) was encoded with Protégé [38] in OWL format, and it is available for download at http://goo.gl/pT1Enz. Based on metrics from Protégé, there are a total of 116 classes, 26 properties (20 object and 6 data). In its current state, the ontology does not utilize any instances, however, we will utilize the ontology to annotate data from various offline and online misinformation sources into the VAXMO model.

We produced some initial scoring to determine an early evaluation (Table 1) of VAXMO’s quality using our in-house web application, OntoKeeper [39, 40]. OntoKeeper is a web-based tool we have developed that calculates metrics rooted in semiotic theory - *semantic*, *pragmatic*, and *syntactic*. These metrics were introduced by Burton-Jones, et al. and have been used in some previous studies to evaluate ontology artifacts [41, 42]. The benefit of this metric according to the authors, is that it is domain independent and applicable to measuring the quality of ontologies of any domain, and concise and easy to interpert and to use for evaluators [43]. OntoKeeper automates the calculations of each of the metrics except for the metrics that involve external participants (i.e. subject matter expert review). The user uploads their ontology and the tools parses and extracts the meta-data needed to calculate the scores and presents them in an easy to use interface. Each of these metrics qualitatively measures the lexical quality of the concept labels (*semantics*), the domain coverage and domain applicability of the ontology (*pragmatic*), the quality of syntax for machine-readability (*syntactic*), and the community usage (*social*). For review of the semiotic evaluation scoring for ontologies see [40, 43] for a primer. As a benchmark, we used the National Center for Biomedical Ontology (NCBO) Bioportal sample evaluation scores from our previous work [40].

**Table 1:** Comparison of quality scoring derived from semiotic metric suite [43] for VAXMO and the NCBO BioPortal sample from [40]

Quality metric	VAXMO	NCBO Sample (*σ*)^a^	z-score
Syntactic	0.69	0.64 (0.14)	0.36
Lawfulness	0.95	0.92 (0.16)	0.19
Richness	0.44	0.36 (0.18)	0.44
Semantic	0.94	0.88 (0.15)	0.40
Interpretability	0.91	0.88 (0.14)	0.21
Consistency	1	0.84 (0.40)	0.40
Clarity	0.95	0.96 (0.13)	-0.08
Comprehensiveness (*Pragmatic*)	< 0.00	0.02 (0.07)	-0.29
Overall Score	0.54	0.51 (0.07)^b^	0.43

^a^scores and values from [40].

^b^Overall score does account for social quality scores reported in [40]

The *syntactic* score, which measures syntax-level assessment of the ontology (i.e. machine readability) based on any breach of syntax (*lawfulness* metric) and utilization of ontology features (*richness* metric) was 0.69, with *lawfulness* and *richness* at 0.95 and 0.44, respectively. The *semantic* score, a score that measures the term label quality of the ontology was rated at 0.94. The *semantic* score is comprised of a *consistency* score that quantifies inconsistent labeling of concepts and instances was 1, *clarity* that quantifies ambiguity of the term labels was 0.95, and *interpretability* that measures the ontology’s term labels’ meaning was 0.91.

For the *comprehensiveness* score (a component of *pragmatic* score to assess the utility of the ontology), we utilized the seed number of 1,277,993, which is the average number of classes, instances, and properties from a sample of NCBO Ontologies in a previous study [40]. Ideally, we would like to have identified appropriate ontologies that are comparable to VAXMO, but for initial scoring we settled on the aforementioned seed number from the previous study. *Comprehensiveness* score from the NCBO seed number provided a very low number value of less than 0.00. The *overall quality* score based on equal weighting of *syntactic* (0.69), *semantic* (0.94), and *pragmatic* (*comprehensiveness* at less than *0.00*) was **0*.*54**. A summary of the scores are presented in Table 1.

We calculated the *z-score* using the data from the NCBO Bioportal scores to attain an initial evaluation. When comparing the *syntactic* score, *z-score* yielded 0.36 indicating above-average syntactic score for VAXMO. The *z-score* for *semantic* was 0.40 also indicating above-average *semantic* score for VAXMO, and the *z-score* for *pragmatic* was −0.29 revealing below-average rating for VAXMO. Also, we calculated the *z-score* for the final *overall quality* using the average NCBO *overall score* (0.51) that does not account for the *social* metric. The z-score for the overall score of VAXMO was 0.43, which is above average in its overall quality compared to the NCBO sample.

We examined the z-score to assess the quality of VAXMO. The *syntactic* score of VAXMO appear to be of higher quality with the NCBO BioPortal sample (z =0.36). We interpreted this to mean that the encoding of the ontology with respect to utilization of formal logic (*richness*) and minimal syntactic violations (*lawfulness*) is better than other ontologies. The semantic score for VAXMO was also better than the sample NCBO BioPortal ontologies (z =0.40) with respect to minimal inconsistencies with the term labels (*consistency*), and with respect to meaningful term labels, i.e. at least one word sense (*interpretability*). However, *clarity* was slightly weaker than average (z =–0.08), where there may have been term labels that had ambiguous meaning, i.e. above average word senses. The sample from NCBO had the benefit of larger ontologies and therefore were more comprehensive in its domain coverage than VAXMO (z =–0.29) in regards to *comprehensiveness*.

Overall, with the exception of *pragmatic* (*comprehensiveness*), the Vaccine Misinformation Ontology (VAXMO) is, in its current state, a relatively respectable quality ontology based on its comparison of *syntactic*, *semantic*, and *overall quality* scores with a sample of NCBO Bioportal ontologies. The low *pragmatic* score indicates the need for greater expansion of the ontology, and we acknowledge that VAXMO still needs some refinement and expansion. In addition, we also plan on attaining a *pragmatic* score’s *accuracy* score [43] that would involve public health experts to provide a review of VAXMO’s veracity which would also produce a more complete *pragmatic* score.

## Theoretical use-cases

Zhou and Zhang have stated that their Misinformation Ontology[37], which is the foundation for VAXMO, could be used for machine-learning tasks to enable machines to detect vaccine misinformation. The features for training would be the classes from the ontology that annotates text, and based on these features potential models can be generated to automatically assess if certain documents or text harbor anti-vaccination opinions. Another future direction is to utilize this ontology to annotate a collection of false statements from the public, specifically in an application-based system where a web-based portal would allow community participants to log statements about vaccines into the system. These false statements would be annotated as nanopublication-types assertions - a benefit of integrating nanopublication - and later be annotated by other concepts of VAXMO to extrapolate features of the false statement. Aside from machine-learning opportunities and application-based usage we may also explore more semantic-based approaches involving natural language processing techniques with ontologies. In the next section we further discuss two use-cases involving machine learning and a method to identify vaccine misinformation in textual content.

In this section, we envision two possible use cases where VAXMO would assist in the detection of vaccine misinformation. One of those use-cases is similar to what has been described in [37], using the ontology to annotate unstructured data. By annotating the data, such as textual information, we can produce a dataset that can be trained by a machine learner. That machine learner would be enabled to reveal statements that contain misinformation. While discussion of machine learning is out the scope of the paper, we introduced a sample of how data can be annotated for machine learning purposes.

### Producing datasets for machine learning

**Fig. 3:**
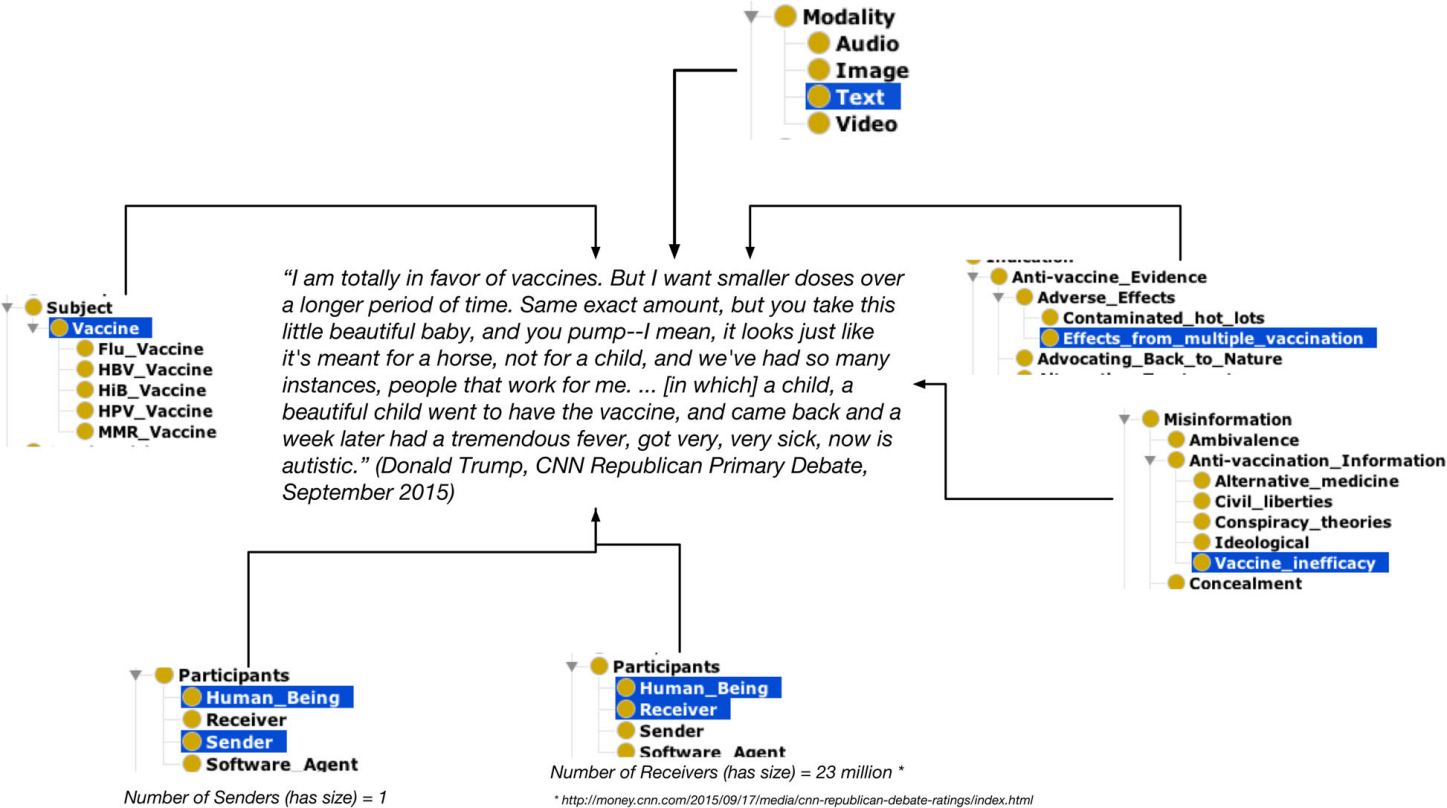
VAXMO for machine learning: Annotating a quote by then-candidate Donald Trump using classes from VAXMO

**Fig. 4:**
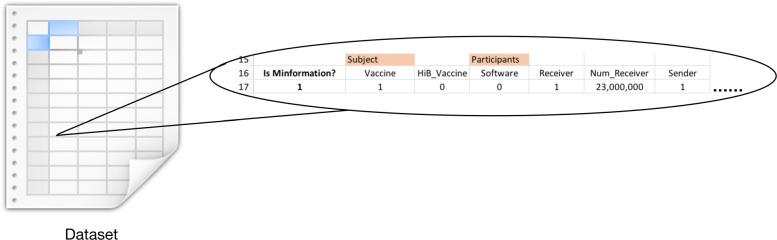
VAXMO for machine learning: Snapshot of the resulting dataset from annotation

Figures 3 and 4 illustrates an example for the aforementioned use-case. Using the classes from VAXMO, one could potentially link the various concepts to unstructured data such as a free text. Figure 3 shows a quote by then-candidate Donald Trump in 2015 stating his position on vaccines. In that example, we demonstrated how some of the various classes (*Subject*, *Modality*, *Anti-vaccine Evidence*, etc.) could be used to annotate the quote. By annotating the data, we can produce a dataset with rows representing whether each class was linked to a piece of data. Figure 4 shows a slice of what the row of data may represent. In the figure, there is a column indicating whether the annotated data is misinformation, followed by each class and subclasses of VAXMO with data designating the features of the annotated data. Determining what to populate into each feature may depend on the type of learner to be used.

While VAXMO might have some possibilities for machine learners, there may be some additional refinement for the ontology needed. One aspect is the ambiguity or fuzziness for a few of the classes. For example, classes like *Availability* with subclass categories of *High Availability* or *Low Availability* may require either some individual estimation, methods to explicitly quantify classes, or adding more categories for further refinement of the concepts. Aside from the ontology itself, the unstructured data may have missing or implied contextual information. While the type of vaccine is not clearly specified in Fig. 3, we may assume the speaker is referring to the MMR vaccine – which in the past has been mistakenly associated with autism. Also, the quote itself does not hint who was spoken to, unless one refers to external references to help provide a link with the *Receiver* class and the number of individuals listening (i.e. for the *hasSize* data property). This is also true of finding out the motive for communicating misinformation to link VAXMO’s *Motivation* concepts. Overall, either finding external references to confirm some of the annotation, or with caution, making an assumption to associate the VAXMO classes with the data may be undesirably necessary for this use-case, but it lends some future work to consider.

### Semantic-driven approach for misinformation detection

Another use-case involves leveraging the triples linked to the ontology through the nanopublication segment of VAXMO. Described earlier, the nanopubulication model for VAXMO was designed to link triples and their meta-data to the overall VAXMO model. VAXMO utilizes nanopublication to link to triples that assert vaccine misinformation which reflect misconceptions permeating some sectors of the general public (e.g. *vaccine causes autism*, *vaccines are utilized to sterilize minority communities*, etc.). For this use-case we applied the use of semi-supervised natural language processing tools to augment the vaccine misinformation triples. For demonstration purposes, we used the description data for a Youtube video discussing some false information about vaccines [44] and the following triples to automatically analyze the video description info:

- *vaccines > causes > seizures*
- *vaccines > results > in death*
- *vaccines > causes > autism*

These above-mentioned triples would be encoded in the assertion line (i.e. line 15 of Listing 1) where each triple would be in their own nanopublication representation.

The sample description text from the Youtube video is:

*Breaking: Doctors Admit Vaccines Cause Convulsions, Brain Damage, And Death In Children. Alex Jones exposes how doctors are fully aware of the adverse side effects of vaccines when administered to children, but the medical community continues to distribute and praise shots.*

To understand the approach for this use-case, we had to define what would constitute misinformation.

#### **Definition 1**

First, we posited that all statements *ST*_*n*_ are either fact *F*_*n*_ or misinformation *M*_*n*_.

1 $$\begin{array}{@{}rcl@{}} \forall \ ST_{n} = F_{n} \oplus M_{n} \end{array} $$

#### **Definition 2**

We presumed that facts and misinformation are composed of ordered tuples of subject *s*, predicate *p*, and objects *o* (i.e. triples).

2 $$\begin{array}{@{}rcl@{}}\forall \ ST_{n} = \left\{\begin{array}{l} \forall \ F_{n} := \langle\ s_{f}, p_{f}, o_{f}\ \rangle \\ \forall \ M_{n} := \langle\ s_{m}, p_{m}, o_{m}\ \rangle \end{array}\right. \end{array} $$

Each subject $ \overline {s} $, predicate $ \overline {p} $, and objects $ \overline {o} $ are a finite string of tokens *e*.

3 $$\begin{array}{@{}rcl@{}} where \ \{ \overline{s}, \overline{p},\overline{o} \} := \{e_{1}e_{2}\dots e_{n} \} \end{array} $$

#### **Definition 3**

Given a statement *ST*, a statement is misinformation *M* where the subject of misinformation triple *s*_*m*_ is similar to the statement’s subject *s*_*st*_, as well as their the predicate *p*_*st*_,*p*_*m*_ and object tuples *o*_*st*_,*o*_*m*_.

4 $$\begin{array}{@{}rcl@{}} ST = M \Rightarrow s_{st} \approx s_{m}\wedge\ p_{st} \approx p_{m}\wedge\ o_{st} \approx o_{m} \end{array} $$

Using this definition (Definition 3), we used the misinformation triples, from VAXMO, to preform matches to identify misinformation of the target statement.

**Fig. 5:**
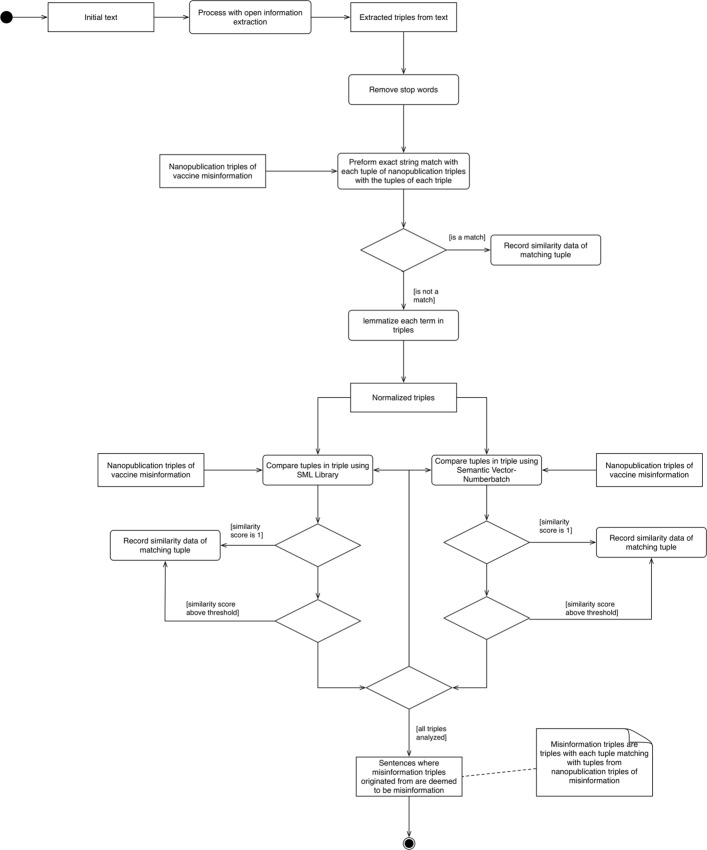
Description of the proof-of-concept method to find vaccine misinformation in text

Figure 5 outlines the method to analyze textual information for misinformation. The entire test of our proof-of-concept method was developed in Java using off-the-shelf natural language processing and semantic web programming libraries. To summarize our process, we initially started with the sample text, and imported the text using an open-sourced open information extraction tool (ClausIE [45]). The exported results were a set of triples from each sentence of the text. The list of triples are provided below.

- *“doctor” > “admit” > “vaccine cause convulsion”*
- *“doctor” > “admit” > “vaccine cause brain damage”*
- *“doctor” > “admit” > “vaccine cause death in child”*
- *“vaccine” > “cause” > “convulsion”*
- *“vaccine” > “cause” > “brain damage”*
- *“vaccine” > “cause” > “death in child”*
- *“alex jone” > “expose” > “how doctor be fully aware of the adverse side effect of vaccine when administer to child”*
- *“doctor” > “be” > “aware” > “fully” > “of the adverse side effect of vaccine” > “when administer to child” > “how”*
- *“the medical community” > “continue” > “to distribute”*
- *“the medical community” > “praise” > “shot”*

We reasoned that stop words may introduce noise in the comparison scoring, so with each of the tuples within the triple, we removed the stop words.

Next, with each triple extracted from the text, we compared the tuples of the triple with the tuples of the misinformation triples from VAXMO using basic exact string matching. If there was an exact match we recorded the match, and if not, we proceeded with the next phase of using graph-based and word-embedding similarity matchings.

Before the next phase, to ensure better accuracy in similarity matching, we lemmatized each term using MorphaStemmer from KnowItAll [46]. After all of the triples were lemmatized, we utilized Semantic Measures Library [47] and ConceptNet Numberbatch term vectors [48] – with Semantic Vectors [49] to interface with the vectors – to compare the similarity of tuples. Noted in our definition, the subject, predicate, and object tuples between the two triples were compared. Any resulting similarity score of the tuples equaling 1 was deemed a match, and any similarity score above a defined threshold would also be deemed a match.

After all triples from the text were analyzed by the code, we assessed the results from the method (See Tables 2, 3, 4, 5, 6, 7, 8, 9, 10 and 11). The first column of scores in each of tables were produced from the Semantic Measures Library (SML) Java library and the second column of scores were produced from Semantic Vectors-Numberbatch (SV-NB). The triples from Tables 2, 3, and 4 appeared to be misinformation, however, none of the three VAXMO triples were similar to the misinformation triples from the text. All of similarity scores were below 0.18 and, therefore, had very low similarity between the tuples.

**Table 2:** Analysis: doctor > admit > vaccine cause convulsion

doctor > admit > vaccine cause convulsion		
vaccines > causes > seizures		
Subject similarity	0.03	0.18
Predicate similarity	0.00	0.00
Object similarity	0.27	0.22
vaccines > results > in death		
Subject similarity	0.03	0.18
Predicate similarity	0.00	0.03
Object similarity	0.05	0.13
vaccines > causes > autism		
Subject similarity	0.03	0.18
Predicate similarity	0.00	0.00
Object similarity	0.11	0.15

**Table 3:** Analysis: doctor > admit > vaccine cause brain damage

doctor > admit > vaccine cause brain damage		
vaccines > causes > seizures		
Subject similarity	0.03	0.18
Predicate similarity	0.00	0.00
Object similarity	0.12	0.12
vaccines > results > in death		
Subject similarity	0.03	0.18
Predicate similarity	0.00	0.03
Object similarity	0.05	0.13
vaccines > causes > autism		
Subject similarity	0.03	0.18
Predicate similarity	0.00	0.00
Object similarity	0.13	0.16

**Table 4:** Analysis: doctor > admit > vaccine cause death in child

doctor > admit > vaccine cause death in child		
vaccines > causes > seizures		
Subject similarity	0.03	0.17
Predicate similarity	0.00	0.00
Object similarity	0.06	0.07
vaccines > results > in death		
Subject similarity	0.03	0.17
Predicate similarity	0.00	0.03
Object similarity	0.31	0.32
vaccines > causes > autism		
Subject similarity	0.03	0.17
Predicate similarity	0.00	0.00
Object similarity	0.05	0.20

**Table 5:** Analysis: vaccine > cause > convulsion

vaccine > cause > convulsion		
vaccines > causes > seizures		
Subject similarity	1.00	1.00
Predicate similarity	1.00	1.00
Object similarity	0.68	0.56
vaccines > results > in death		
Subject similarity	1.00	1.00
Predicate similarity	0.50	0.44
Object similarity	0.04	0.13
vaccines > causes > autism		
Subject similarity	1.00	1.00
Predicate similarity	1.00	1.00
Object similarity	0.20	0.12

**Table 6:** Analysis: vaccine > cause > brain damage

vaccine > cause > brain damage		
vaccines > causes > seizures		
Subject similarity	1.00	1.00
Predicate similarity	1.00	1.00
Object similarity	0.17	0.19
vaccines > results > in death		
Subject similarity	1.00	1.00
Predicate similarity	0.50	0.44
Object similarity	0.04	0.12
vaccines > causes > autism		
Subject similarity	1.00	1.00
Predicate similarity	1.00	1.00
Object similarity	0.20	0.16

**Table 7:** Analysis: vaccine > cause > death in child

vaccine > cause > death in child		
vaccines > causes > seizures		
Subject similarity	1.00	1.00
Predicate similarity	1.00	1.00
Object similarity	0.04	0.08
vaccines > results > in death		
Subject similarity	1.00	1.00
Predicate similarity	0.50	0.44
Object similarity	0.56	0.51
vaccines > causes > autism		
Subject similarity	1.00	1.00
Predicate similarity	1.00	1.00
Object similarity	0.04	0.22

**Table 8:** Analysis: alex jone > expose > how doctor be fully aware of the adverse side effect of vaccine when administer to child

alex jone > expose > how doctor be fully aware of the adverse side effect of vaccine when administer to child		
vaccines > causes > seizures		
Subject similarity	0.00	0.00
Predicate similarity	0.10	0.21
Object similarity	0.06	0.06
vaccines > results > in death		
Subject similarity	0.00	0.00
Predicate similarity	0.10	0.12
Object similarity	0.04	0.04
vaccines > causes > autism		
Subject similarity	0.00	0.00
Predicate similarity	0.10	0.21
Object similarity	0.05	0.11

**Table 9:** Analysis: doctor > be > aware > fully > of the adverse side effect of vaccine > when administer to child > how ^a^ compares the highest similarity score of the multiple arguments after the predicate with the target object of the predicate

doctor > be > aware > fully > of the adverse side effect of vaccine > when administer to child > how		
vaccines > causes > seizures		
Subject similarity	0.04	0.17
Predicate similarity	0.00	0.00
Object similarity^a^	0.05	0.11
vaccines > results > in death		
Subject similarity	0.04	0.17
Predicate similarity	0.00	0.00
Object similarity^a^	0.05	0.07
vaccines > causes > autism		
Subject similarity	0.04	0.17
Predicate similarity	0.00	0.00
Object similarity^a^	0.02	0.19

**Table 10:** Analysis: the medical community > continue > to distribute

the medical community > continue > to distribute		
vaccines > causes > seizures		
Subject similarity	0.04	0.08
Predicate similarity	0.00	0.16
Object similarity	0.00	0.09
vaccines > results > in death		
Subject similarity	0.04	0.08
Predicate similarity	0.00	0.25
Object similarity	0.00	0.00
vaccines > causes > autism		
Subject similarity	0.04	0.08
Predicate similarity	0.00	0.16
Object similarity	0.00	0.00

**Table 11:** Analysis: the medical community > praise > shot

the medical community > praise > shot		
vaccines > causes > seizures		
Subject similarity	0.04	0.08
Predicate similarity	0.10	0.00
Object similarity	0.27	0.02
vaccines > results > in death		
Subject similarity	0.04	0.08
Predicate similarity	0.10	0.02
Object similarity	0.04	0.06
vaccines > causes > autism		
Subject similarity	0.04	0.08
Predicate similarity	0.10	0.00
Object similarity	0.21	0.00

Tables 5, 6 and 7 showed some identification of misinformation through our test method. *vaccine > cause > convulsion* revealed to be similar to the VAXMO triple of *vaccines > causes > seizures* (Table 5). Both the subject and predicate tuples were highly similar with a score of 1.00, and object similarity comparing *convulsion* and *seizures* were above 0.68 (SML) and 0.56 (SV-NB). With results in Table 6, we assumed that *vaccine > cause > brain damage* would be approximatively similar to *vaccines > causes > autism*, but unfortunately this did not succeed. Both their subject and predicate tuples were highly matched, but the similarity analysis revealed that *brain damage* and *autism* were not similar, with scores of 0.20 (SML) and 0.16 (SV-NB). Same as Table 5, Table 7’s data revealed some success in identifying misinformation – *vaccine > cause > death in child* were similar to *vaccines > results > in death*. The subject tuples were a match, and the predicate and object comparison had high similarity scores. The SV-NB score for the predicate comparison was 0.44 but the SML score was at 0.50. Object similarity was 0.56 (SML) and 0.51 (SV-NB).

For the remaining data, none of the triples from the text appear to have vaccine misinformation, or were relevant by our observation. Tables 8 through 11 are provided for examination purposes.

The approach described in this subsection is a proof-of-concept method, yet there are some limitations to this method. One such limitation is that we need to be aware and encode vaccine misinformation beforehand into VAXMO. In the sample test, there was a possible false statement mentioning that doctors admit vaccine causes harmful effects. If we wanted to denote that it is misinformation we would need a triple in VAXMO that expressed that notion. Another limitation was determining a threshold. In one example we noted that similar tuples had at least 0.50 similarity score. However, we assumed that future examples, when we further test this method, may yield similarity scores below 0.50. Generally, we would need to identify a minimal threshold that would maximize the effectiveness of this method to identify misinformation. Lastly, as VAXMO’s misinformation triples grows in number or if there extensive number of triples in a document or text, we would need to assess if this method is scalable and determine if it would perform relatively fast. Overall, testing this proof-of-concept method is needed on various pieces of text for future research endeavors.

## Discussion and conclusion

The Vaccine Misinformation Ontology (VAXMO)’s purpose is to catalogue and analyze vaccine misinformation that has been one of the drivers for low rates of vaccination rates worldwide. Ontologies benefit from reusing other ontologies. We have utilized an existing model of misinformation (Misinformation Ontology) to address anti-vaccination information. In addition, we have utilized an innovative approach using nanopublication (which is generally used for scientific assertions) for linking common false assertions or theories about vaccines (i.e. “vaccines causes autism”, “government created weaponized Ebola vaccines”, etc.). Yet, this poses some difficulty - lack of Protégé support and manually editing the ontology artifact. This may inspire us to investigate the possibility of developing a Protégé plugin that provides an interface to view and edit the nanopublication segment of VAXMO.

With some modifications, we constructed the ontology based off of the Misinformation Ontology and extended some of its concepts from an existing survey literature. While MO is specifically designed to model false intention and not misfacts, as stated by the original authors, we further extended the ontology to utilize nanopublication graph structure to store and represent false assertions about vaccines. The current representation of VAXMO is encoded in OWL with only the class-level fleshed out and with some conceptual gaps.

Noted earlier, there have been various studies that focused on content analysis of misinformation and myths of vaccines in the public health domain. Some of the literature can help furnish additional concepts to further expand VAXMO, which could help model and understand the features within anti-vaccination information domain.

While VAXMO is of better quality than NCBO Bioportal ontologies, there is still some more work needed to expand its conceptual domain space for anti-vaccine information. Also, we have described a future use-case that aims to detect misinformation about vaccines, and we plan on reporting on our findings in a future study.

We assume that the impact of this work could lead to applicable uses of semantic web ontologies for public health informatics and future informatics tools that can assist researchers to understand and address health misinformation in the post-modern era.

## Abbreviations

MO: Misinformation ontology
NCBO: National center for biomedical ontology
OWL: Web ontology language
RDF: Resource description framework
VAXMO: Vaccine misinformation ontology
